# Alginate Microencapsulation of Human Islets Does Not Increase Susceptibility to Acute Hypoxia

**DOI:** 10.1155/2013/374925

**Published:** 2013-12-01

**Authors:** I. K. Hals, A. M. Rokstad, B. L. Strand, J. Oberholzer, V. Grill

**Affiliations:** ^1^Department of Cancer Research and Molecular Medicine, Faculty of Medicine, Norwegian University of Science and Technology, Postbox 8905, 7491 Trondheim, Norway; ^2^Department of Biotechnology, Faculty of Natural Sciences and Technology, Norwegian University of Science and Technology, 7034 Trondheim, Norway; ^3^Department of Surgery, University of Illinois, IL at Chicago, Chicago, IL 60612, USA; ^4^Department of Endocrinology, St. Olavs Hospital, Trondheim University Hospital, Postbox 3250, 7006 Trondheim, Norway

## Abstract

Islet transplantation in diabetes is hampered by the need of life-long immunosuppression. Encapsulation provides partial immunoprotection but could possibly limit oxygen supply, a factor that may enhance hypoxia-induced beta cell death in the early posttransplantation period. Here we tested susceptibility of alginate microencapsulated human islets to experimental hypoxia (0.1–0.3% O_2_ for 8 h, followed by reoxygenation) on viability and functional parameters. Hypoxia reduced viability as measured by MTT by 33.8 ± 3.5% in encapsulated and 42.9 ± 5.2% in nonencapsulated islets (*P* < 0.2). Nonencapsulated islets released 37.7% (median) more HMGB1 compared to encapsulated islets after hypoxic culture conditions (*P* < 0.001). Glucose-induced insulin release was marginally affected by hypoxia. Basal oxygen consumption was equally reduced in encapsulated and nonencapsulated islets, by 22.0 ± 6.1% versus 24.8 ± 5.7%. Among 27 tested cytokines/chemokines, hypoxia increased the secretion of IL-6 and IL-8/CXCL8 in both groups of islets, whereas an increase of MCP-1/CCL2 was seen only with nonencapsulated islets. *Conclusion*. Alginate microencapsulation of human islets does not increase susceptibility to acute hypoxia. This is a positive finding in relation to potential use of encapsulation for islet transplantation.

## 1. Introduction

Transplantation of pancreatic islets containing the insulin-producing beta cells could in principle cure type 1 diabetes. However, transplantation of allografts necessitates treatment with immunosuppressant drugs with ensuing side effects. Encapsulation of islets (or isolated beta cells) could potentially alleviate this problem, thus motivating previous and ongoing research on the feasibility of encapsulated islets for successful transplantation. Promising results (reversal of diabetes in animal models of diabetes) have been reported [1–4]. However, questions remain as to both the short and long term functionality of encapsulated islets or beta cells.

One question pertains to the impact of hypoxia on encapsulated islets. Native pancreatic beta cells have high rates of oxidative metabolism to meet the demand of insulin production and secretion [5], and even moderately decreased levels of oxygen have been shown to inhibit insulin release [6]. Hypoxia after transplantation is a major (albeit not the only) contributor to the dramatic drop of viable beta cells (nonencapsulated) that occurs in the immediate period following transplantation [7–10]. A negative impact of the—inevitable—hypoxia during the immediate period following transplantation could possibly be worsened by encapsulation, since the distance of diffusion for oxygen could be greater in encapsulated versus nonencapsulated islets (or amassed beta cells) [11, 12], and a negative effect of clustering of islets may occur [13]. Comparisons of oxygen uptake in encapsulated versus nonencapsulated islets have been done for neonatal porcine [14] and pig [15] islets *in vitro* (normoxic conditions) without unveiling negative effects of encapsulation, while encapsulation of rat islets led to a significant reduction of oxygen uptake [16]. However, a similar comparison has, to the best of our knowledge, not been made for human islets, neither in a setting of normoxic nor hypoxic culture conditions.

The aim of the present study was to compare viability and functional parameters of encapsulated versus nonencapsulated islets during normoxic culture conditions and in particular after a defined period of hypoxia. We chose an *in vitro* approach, since testing could be influenced by site for transplantation, thereby modifying a basic impact of hypoxia. We used alginate microbeads, since such a preparation has recently been shown to be a promising candidate for immune protection in light of its low inflammatory potential [17, 18] as well as functional performance in mice models [1, 2]. A recent study using similar microbeads highlights beneficial effects of encapsulation on human islet functionality [4].

## 2. Materials

Ultrapure sodium alginate from *Laminaria hyperborea*, Pronova UP-LVG (67% guluronic acid, viscosity 1051 mL/g, endotoxin 23 EU/g, MW 187 × 10^3^) was from Nova-Matrix, Oslo, Norway. Other materials were from Sigma-Aldrich Chemicals Co. (St. Louis, MO) or from sources specified below.

## 3. Methods

### 3.1. Islet Procurement, Isolation, and Shipment

Human islets were isolated at the Division of Transplant, University of Illinois, Chicago, as previously described [19, 20] and shipped to Trondheim. Sustained function of shipped human islets has previously been documented [1]. The purity and viability (determined on the basis of dithizone staining) and insulin secretion at the time of shipment as well as donor characteristics are presented in Table 1. Totally 5 shipments were received, each shipment containing islets from a single donor. The Regional Committee for Medical and Health Research Ethics Central, Norway, approved the procurement of human islets and their use for research. Only islets from donors with research consent were used.

### 3.2. Islet Culture and Microencapsulation

Upon arrival in Trondheim, islets were centrifuged (1000 rpm, 2 min) and resuspended in RPMI 1640 medium containing 5.5 mM glucose and supplemented with 10% fetal calf serum (FCS), 10 mM HEPES, 4 mM L-glutamine, 1 mM sodium pyruvate, 100 IU/mL penicillin, and 100 *μ*g/mL streptomycin. Islets were cultured in flasks at 37°C in a humidified atmosphere of 5% CO_2_ in air.

The number of islets was estimated after overnight culture. One half of the islets was then encapsulated in alginate, while the other half remained nonencapsulated. For encapsulation, 10–20 000 islet equivalents were centrifuged (1000 rpm, 2 min) and resuspended in 300–400 *μ*L of RPMI with 5.5 mM glucose. They were then added to 1.8 mL of 2.0% sterile filtered UP-LVG alginate (in 0.3 M mannitol, pH 7.3). Inhomogeneous alginate microbeads were formed by use of an electrostatic bead generator (7 kV, one 0.4 mm needle, flow 10 mL/h) using a gelling solution of 50 mM CaCl_2_, 1 mM BaCl_2_, 150 mM mannitol, and 10 mM HEPES, at pH 7.3. The microbeads (521 ± 10 *μ*m in diameter, measurements from 33 capsules, each containing 1–4 islets) were collected on a filter and then washed three times with 12 mL of Hank's and once with 20 mL of culture media before being transferred to a culture flask. Both encapsulated and nonencapsulated islets were cultured overnight and used for experiments 1–23 days later. We did not observe clumping of nonencapsulated islets to any major extent. Further, we did not detect obvious differences due to length of culture on function and viability neither in encapsulated nor in nonencapsulated islets.

### 3.3. Experimental Protocols

Prior to each experiment, aliquots from homogenous suspensions of encapsulated and nonencapsulated islets were collected to estimate the number of islets per mL culture media.

Encapsulated and nonencapsulated islets were divided into each of two groups and transferred to Petri dishes (with <100 islets per 5 mL culture medium) before exposure to either continuous normoxia or to 8 h of hypoxia followed by 14–18 h of reoxygenation. Hypoxia was induced by placing islets in a hypoxia chamber (Billups-Rothenberg Inc., Del Mar, CA) together with an oxygen monitor (Dräger Safety AG & Co., KGaA, Lübeck, Germany) and a Petri dish with 5–10 mL of water for humidity. The chamber interior was flushed with nitrogen gas (95% N_2_, 5% CO_2_) until a level of 0.1% O_2_ was reached. After 8 hours of incubation the oxygen concentration inside the chamber had risen to 0.2-0.3%.

For each of the four different experimental conditions (i.e., normoxia or hypoxia for encapsulated and normoxia or hypoxia for nonencapsulated islets) a control estimate of islet number per dish was made also after the end of the reoxygenation period. Islets from the different culture conditions, as well as aliquots of culture media, were sampled for measurements as detailed below.

### 3.4. MTT

For each experimental condition, 30 or 40 islets (encapsulated as well as nonencapsulated) were handpicked into each of 2–5 parallel wells on a 24-well plate for 4 h of exposure to 3-(4,5-dimethyl-2-thiazolyl)-2,5-diphenyltetrazolium bromide (MTT). The MTT reagent in the media was removed by washing the islets several times in 0.9% NaCl. Islets were then incubated for one hour in 400 *μ*L DMSO per well at 37°C for color development. Fifty *μ*L/well of 0.1 M NaCl in 0.1 M Glycine, pH 10.5, was added for color extraction. Two parallel aliquots per well were secured for absorbance measurements.

### 3.5. HMGB1

The amount of high mobility group box 1 (HMGB1) in media aliquots (kept at −80°C until assay) was measured by a HMGB1 ELISA kit (IBL International, Hamburg, Germany). The assay was performed as recommended by the producer.

### 3.6. Insulin Secretion

Groups of 5 handpicked islets were placed in each of 5-6 parallel wells in a 24-well plate. This was followed by preincubation for 30 min at 37°C in 0.5 mL of Krebs-Ringer bicarbonate buffer (KRB, containing 0.5% BSA and 10 mM HEPES at pH 7.4) together with 1.6 mM glucose. Islets were transferred into a new 24-well plate and incubated for another 60 min in KRB containing 1.6 mM glucose in order to assess basal (= un-stimulated) insulin secretion. The same islets were finally transferred to a new 24-well plate for stimulated insulin secretion by incubation for 90 min in KRB with 16.7 mM glucose. Aliquots of incubation media were secured for basal as well as stimulated secretion. Samples were kept at −20°C pending insulin measurements by a RIA kit for human insulin (Millipore, St. Charles, MO).

### 3.7. Oxygen Consumption

Equal amounts of islets were transferred to each of three dishes and exposed to hypoxia for 8 h followed by 14–18 h of reoxygenation. Control islets were cultured continuously at normoxia. For each condition islets (3x approx. 300 islets) were pooled into one sample immediately before the oxygen consumption measurements.

Oxygen consumption was measured by Clark-type polarographic oxygen sensors and high-resolution respirometry (Oxygraph-2k, OROBOROS, Innsbruck, Austria). Samples of up to 900 islets in culture medium (corresponding to ~10^6^ islet cells/cm^3^) were added to a chamber. Islets were allowed to sediment before closing the chamber, switching on magnetic stirring and recording oxygen uptake at basal respiration. The ATP synthase inhibitor oligomycin (2 *μ*g/mL) was subsequently added with the aim to assess uncoupled (= not ATP-coupled) respiration. After that, the protonophore carbonyl cyanide p-trifluoromethoxyphenylhydrazone (FCCP) was added and titrated (up to 5-6 *μ*M) to achieve a state of maximum respiratory capacity. Finally, rotenone (0.5 *μ*M) and antimycin (2.5 *μ*M), inhibitors of mitochondrial complexes I and III, were added in order to measure residual oxygen consumption (ROX). Oxygen consumption rates were calculated as the negative time derivate of the oxygen concentration present in the chamber (pmol/s/mill cells). For all experiments, ROX values were close to zero.

### 3.8. Cytokine/Chemokine Measurements

For all islet culture conditions, aliquots of the culture media were harvested after the reoxygenation period. For a subset of experiments, aliquots of media were secured also immediately after the time of hypoxia exposure. Samples were kept at −80°C until assays were performed.

Islet secreted mediators were analyzed by a multiplex bead-based cytokine assay (Bio-Plex Human Cytokine Group I 10-Plex Panel, Bio-Rad Laboratories, Hercules, CA) containing the following analytics: IL-1ra, IL-6, IL-8/CXCL8, IL-9, IL-10, IL-12(p70), GM-CFS, MCP-1/CCL2, MIP-1*β*/CCL4, and VEGF. In addition, we included Bio-Plex kit reagents for detecting MIF, a part of Human Cytokine Group II. Our panel of mediators was chosen on the basis of preanalyzed samples (from each of the four conditions) using a Bio-Plex Human Cytokine Group I 27-plex panel (Bio-Rad Laboratories, Hercules, CA). The following analytics were below the detection limit and therefore excluded from the main analyses; IL-1*β*, IL-2, IL-4, IL-5, IL-13, IL-15, IL-17, Eotaxin/CCL11, Basic FGF, IFN-*γ*, IP-10/CXCL10, MIP-1*α*/CCL3, PDGF-BB, Rantes/CCL5, and TNF-*α*. The multiplex analyses were performed as recommended by the producer except for using half the recommended amounts of beads. For intra-assay variations, see Supplementary Table S1 of the Supplementary Material available online at http://dx.doi.org/10.1155/2013/374925.

### 3.9. Microbead Dissolution, Extraction, and Measurement of DNA

To assess DNA contents microcapsules first had to be completely dissolved. Dissolving of alginate microbeads (350 *μ*L) was achieved by adding 1000 *μ*L of tetra sodium EDTA (50 mM, pH 8) at 37°C followed by intermittent vortexing for up to 30 minutes. After centrifugation (10 min, 13000 rpm), the islet pellet was harvested and resuspended in water for extraction of DNA. Nonencapsulated islets were extracted by the same procedure. DNA was quantified by the Fluorescent DNA Quantification kit (Bio-Rad, Hercules, CA).

## 4. Statistics

Data are presented as mean ± SEM. Also medians were calculated. The Wilcoxon rank test was used for significance testing. A *P* value < 0.05 was defined as statistically significant.

## 5. Results

### 5.1. MTT

Islet viability assessed by MTT is presented in Table 2. The mean absorbance values were identical for encapsulated and nonencapsulated islets after continuous normoxia. Previous hypoxia exposure significantly reduced the MTT parameter of viability in both groups of islets by 33.8 ± 3.5% versus 42.9 ± 5.2% (*P* < 0.2 for difference). There was thus no tendency for a stronger effect of hypoxia in the encapsulated islets.

### 5.2. HMGB1

Hypoxia-induced islet damage has been associated with HMGB1 release [21, 22]. The release of HMGB1 was therefore used as a marker for islet destruction. Compared to encapsulated islets, nonencapsulated islets released 22.4 ± 13.3% more (*P* < 0.2) HMGB1 under continuous normoxia. Levels of HMGB1 were significantly increased in media from both groups of islets after experimental hypoxia by 37.2 ± 15.2% (median: 35.0%) for encapsulated and by 39.7 ± 28.7% (median: 33.3%) for nonencapsulated islets (*P* < 0.2 for difference, *n* = 13). However nonencapsulated islets released in total 43.1 ± 9.3% (median: 37.7%) more HMGB1 than encapsulated islets under hypoxic culture conditions (*P* < 0.001, *n* = 13).

### 5.3. Insulin Secretion

**Figure 1:**
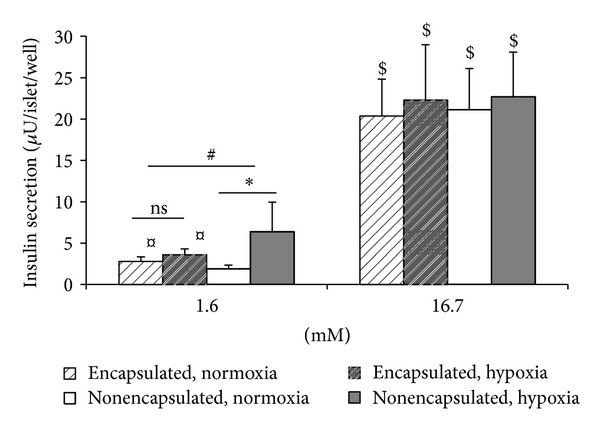
Effect of hypoxia and encapsulation on insulin secretion at 1.6 and 16.7 mM glucose. ^$^ *P* < 0.02 for the stimulatory effect of 16.7 mM glucose, *P < 0.02 for the effect of hypoxia, ^¤^ *P* < 0.04 for the effect of encapsulation, and ^#^ *P* < 0.02 for the effect of hypoxia on encapsulated versus nonencapsulated islets at basal secretion. Data are mean ± SEM of eight separate experiments (five-six parallels per experimental condition), one-three experiments per donor (four donors).

Insulin release during normoxia and low glucose (1.6 mM) was modest (in comparison to stimulated insulin release) for both encapsulated and nonencapsulated islets (Figure 1). Secretion in this unstimulated state was somewhat higher in encapsulated versus nonencapsulated islets conditions (*P* < 0.04). During the same conditions of oxygen supply (normoxia, no previous hypoxia) raising the glucose concentration to 16.7 mM elicited a strong (10–14-fold) insulin response in both types of islet preparations. The fold increase due to 16.7 mM glucose, named the glucose stimulation index (GSI), was lower in encapsulated than in nonencapsulated islets (10.0 ± 3.1 versus 15.9 ± 4.7, *P* < 0.04). Interestingly, the GSI after shipment and culture in Trondheim for various times was higher than GSI after isolation, as recorded in Table 1.

After exposure to hypoxia, insulin secretion at low glucose concentrations increased in nonencapsulated islets (*P* < 0.04) but not in encapsulated islets (Figure 1). Hypoxia did not affect the stimulatory effect of 16.7 mM glucose in absolute terms of insulin secretion whether in nonencapsulated or encapsulated islets. However, due to the increased insulin secretion during low glucose, the hypoxia-induced reduction of the GSI was more pronounced in nonencapsulated islets (68.7 ± 6.1%, from 15.9 ± 4.1 to 3.9 ± 0.9, *P* < 0.02) than in encapsulated islets and (24.5 ± 11.2%, from 10.0 ± 3.1 to 6.3 ± 1.1, ns).

### 5.4. Oxygen Consumption

**Figure 2:**
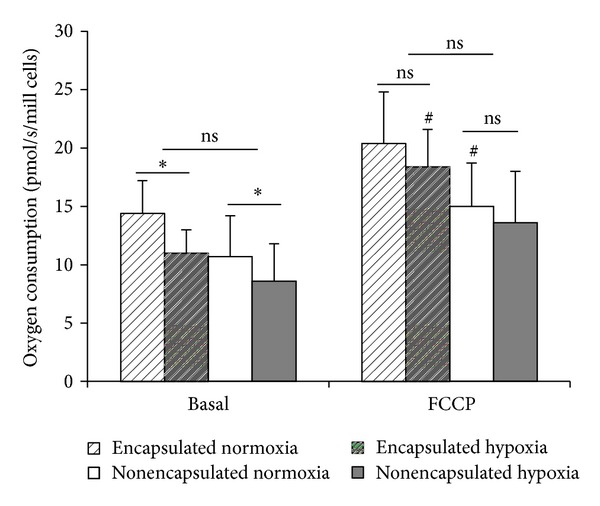
Effect of hypoxia and encapsulation on oxygen consumption. *P < 0.05 for the effect of hypoxia and ^#^ *P* < 0.05 for the effect of FCCP versus basal respiration. Data are mean ± SEM of five-six separate experiments (one-three parallels per experimental condition), one-two experiments per donor (four donors).

The oxygen consumption in encapsulated and nonencapsulated islets following culture under normoxia or transient hypoxia is shown in Figure 2. At basal conditions after continuous normoxia oxygen consumption appeared slightly but not significantly higher in encapsulated versus nonencapsulated islets. Exposure to hypoxia reduced oxygen consumption both in encapsulated (by 22.0 ± 6.1%) and nonencapsulated islets (by 24.8 ± 5.7%, *P* < 0.7 for comparison). Compared to basal respiration, addition of FCCP (an inducer of maximal respiration capacity) led to a modest increase in oxygen uptake for islets subjected to all four culture conditions. Hypoxia tended to reduce FCCP-induced respiration (by 9.7 ± 13.1% in encapsulated and by 2.9 ± 6.9% in nonencapsulated islets, *P* < 0.3 for comparison) (Figure 2).

The lack of a “plateau” of oxygen uptake following the administration of oligomycin A (results not shown) hampered a more detailed analysis of the impact of hypoxia on oxygen consumption.

Islet DNA contents were measured in two representative experiments. Encapsulated islets contained a mean of 3.19 *μ*g DNA/sample and nonencapsulated islets 2.31 *μ*g DNA/sample. Corresponding values obtained after hypoxia were 2.63 and 2.12 *μ*g DNA/sample. These differences in DNA content parallel the slightly (ns) higher basal oxygen uptake seen in encapsulated versus nonencapsulated islets in Figure 2 (+19.8 ± 12.8% for normoxia and +13.6 ± 17.2% for hypoxia).

### 5.5. Cytokine/Chemokine Secretion

**Figure 3:**
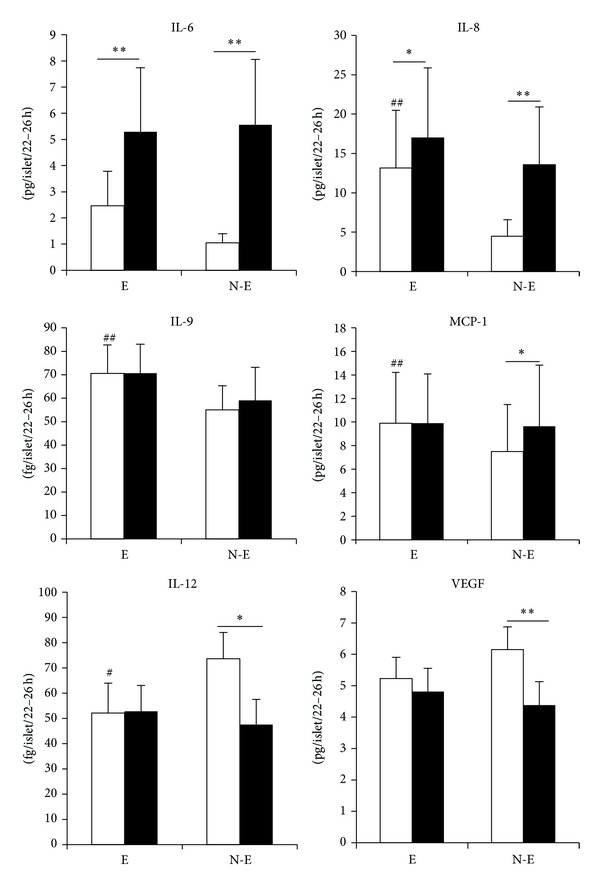
Secreted mediators from encapsulated (E) and nonencapsulated (N-E) islets following culture in continuous normoxia (open bars) and transient hypoxia (filled bars). *P < 0.03 and **P < 0.001 for the effect of hypoxia, ^#^ *P* < 0.05 and ^##^ *P* < 0.003 for the effect of encapsulation during normoxia. Data are mean ± SEM of 13 separate experiments (one sample per condition), one-five experiments per donor (five donors).

The most secreted mediators detected by the multiplex analysis were IL-6, IL-8/CXCL8, MCP-1/CCL2, IL-9, IL12, and VEGF. The secretion profiles of these mediators from encapsulated and nonencapsulated islets after culture under normoxic and transient hypoxic conditions are given in Figure 3. The accumulation (pg or fg/islet/22–26 h) of IL-8/CXCL8, IL-9, and MCP-1/CCL2 was significantly increased from encapsulated versus nonencapsulated islets under basic normoxic conditions. The opposite tendency (reduced accumulation for encapsulated versus nonencapsulated islets) was seen for IL-12 (significant) and VEGF (nonsignificant). Experimental hypoxia significantly enhanced the accumulation of IL-6 and IL-8/CXCL8 for both groups of islets, whereas the secretion of MCP-1/CCL2 was significantly increased from nonencapsulated while unchanged from encapsulated islets. Hypoxia did not affect the accumulation of IL-9 from neither group of islets. The secretion of IL-12 and VEGF was significantly reduced from nonencapsulated islets and unchanged for encapsulated islets.

The ratios of hypoxia-induced accumulation of cytokines/chemokines (H) divided by the accumulation during continuous normoxia (N) (H/N ratio) are summarized in Table 3. Notably, the hypoxia-induced enhancement or reduction of presented mediators was less pronounced for encapsulated islets versus nonencapsulated islets. Hence, the H/N ratios were closer to unity for encapsulated islets.

Levels of IL-1ra, IL-10, GM-CSF, MIP-1*β*/CCL4, and MIF were close to the detection limit. These data are presented as Supplementary Figure S1 and Table S2. Significant effects of encapsulation were seen during basic conditions for MIP-1*β*/CCL4 and MIF (increased secretion) and IL-10 (decreased).

We also wished to address the question whether hypoxia-induced changes/alterations of the accumulation of cytokines take place mainly during the hypoxic event or following hypoxia (i.e., during reoxygenation). We compared in a subset of experiments, *n* = 4 (representing two experiments for each of two donors/islet isolates), the accumulation during the 8 h of hypoxia with that which was secreted during the hypoxia + reoxygenation period. The data presented in Supplementary Table S3 show that the secretion of IL-6, IL-8/CXCL8, MCP-1/CCL2, and VEGF continue to a large extent during the reoxygenation period. The most pronounced lingering effect of hypoxia was seen for IL-6. From nonencapsulated islets almost 90% was secreted during the time period of reoxygenation. Secretion differences between the hypoxia and the reoxygenation period tended to be less pronounced for encapsulated islets (Supplementary Table S3 and Supplementary Table S4). For IL-9 and IL-12 the secreted levels after 8 h of hypoxia were hardly detectable.

## 6. Discussion

Our results indicate, in general, that the impact of acute and severe hypoxia is no more negative for microencapsulated than for nonencapsulated human islets whether in terms of viability or function. In fact the effects of hypoxia on some parameters (MTT, HMGB1, and cytokines) were less, or tended to be less, marked in the encapsulated islets. The relevance of these findings would appear strengthened by the human islets being of high purity and displaying at the onset of experiments good function in terms of glucose-induced insulin secretion and oxygen consumption.

HMGB1 is secreted from human islets as a response to islet damage [21] and is recently shown to be secreted during hypoxia [22]. Our data confirm increased levels of HMGB1 secretion after hypoxic culture conditions, a finding shared by encapsulated and nonencapsulated human islets. However, we find less total secretion of HMGB1 from encapsulated versus nonencapsulated islets after hypoxia exposure. This suggests that the microcapsule could have a protective effect against islet destruction. As a further implication, one may note that HMGB1, due to its inflammatory properties of [23, 24], might be a contributor to the fibrotic responses against encapsulated islets, such responses being a major challenge in islet encapsulation based therapy. Also in this respect the lower secretion of HMGB1 from encapsulated islets could possibly be viewed as a positive finding. It should however be emphasized that the direct impact of HMGB1 in human islet transplantation has recently been questioned [21].

The insulin data point to subtle effects of encapsulation and in particular a slightly increased secretion during basal normoxic conditions. The cause(s) behind this difference has not been elucidated. Based on the hypoxia-induced rise in basal insulin release that we observe for nonencapsulated islets, one possible explanation could be that islets within the microcapsule are subjected to mild hypoxia, which subsequently gives rise to the basal secretion. Data from Vaithilingam et al. could support such notion since pretreatment with the hypoxia-mimicking desferrioxamine agent induces slightly elevated basal insulin secretion [25].

A high degree of oxygen consumption recorded *in vitro* has been linked to successful transplantations [26–28]. It is thus a positive finding (in terms of the clinical utility of encapsulated islets) that oxygen consumption under basic conditions (no hypoxia) was not diminished in encapsulated islets (Figure 2). These findings are in line with those reported previously for pig islets encapsulated in a monolayer cellular device [15] and for microencapsulated neonatal porcine islets [14]. The recorded values of oxygen flux (pmol/s/mill cells) in our study are comparable to the findings for rat islets by use of the same type of oxygraph [29]. As to the hypoxia-induced decrease in oxygen consumption that we find, this was anticipated from previous studies on nonencapsulated rat islets [30]. Importantly, the decrease in oxygen consumption due to hypoxia was not aggravated by microencapsulation.

The production and secretion of cytokines and chemokines are thought to reflect, in part at least, the impact of stressors on islets [31]. Cytokines and chemokines are of general importance in graft destruction due to their various roles in inflammation, angiogenesis, and microcapsule fibrosis. It was therefore of interest to compare the secretion of these biologically active substances from encapsulated and nonencapsulated islets.

Of the cytokines/chemokines that could be reliably measured, we found that IL-6, IL-8/CXCL8, IL-9, and MCP-1/CCL2 were increased, or tended to be, by encapsulation, whereas IL-12 and VEGF tended to be decreased. IL-6 is generally known as a strong proinflammatory cytokine but is also shown to have anti-inflammatory properties [32, 33]. Various regulatory roles of immune responses are also found for IL-9 [34] and IL-12 is an important regulator of helper T cells [35]. IL-8/CXCL8 is a strong attractor for neutrophils [36] and contributes to angiogenesis [37]. VEGF is a key proangiogenic growth factor [38, 39]. MCP-1/CCL2 is an important chemokine in attraction of monocytes and has been negatively associated to graft function [40].

It should be noted that secretion of VEGF was decreased in response to hypoxia rather than increased. At first glance this finding in nonencapsulated islets is paradoxical, since treatment of human islets with the hypoxia-mimicking agent desferrioxamine increased the expression of VEGF [25]. However, a study with islets indicated that a 48 h culture in normoxia increased VEGF mRNA and protein to an extent that further increase was not observed after hypoxia [41]. Possibly, cells in the core of hypoxic islets have been damaged to an extent which could have impaired their production and secretion of VEGF. Also, our finding is in line with a previous paper showing reduced secretion of VEGF in response to a different stressor, that is, enterovirus infection [42]. It seems clear that the regulation of VEGF by different stressors under different experimental conditions needs to be studied further.

In general, the hypoxia-induced enhancement of cytokine/chemokine secretion appeared less marked from encapsulated islets. This could, in part at least, be secondary to the somewhat higher secretion during normoxic (no previous hypoxia) conditions. Whether this indicates that encapsulation induces more stress during basic conditions of culture cannot be decided. Speaking against such notion are the viability, oxygen consumption, and insulin data. In any case the data provided here do not indicate enhanced cytokine/chemokine responses to hypoxia in encapsulated versus nonencapsulated islets.

There are limitations to the study. With islets from a larger number of donors one could possibly have detected additional subtle differences between encapsulated and nonencapsulated islets. Further, one should acknowledge that we do not know which cells in the islet preparations that actually release the cytokines/chemokines. For IL-8/CXCL8 it has been shown that this cytokine can be produced by human *β*-cells [31]. Yet, clinically this uncertainty may be of less importance, since all cells that are part of an islet preparation will be transplanted. Also different encapsulation protocols have been used in previous studies, and we did not test for potential effects of such differences in the present context. However, the composition of alginate employed here has been widely used in recent years and transplantations of islets in similar microcapsules have given promising results in animal models of diabetes [1, 2, 4], and future clinical studies with such capsules seem clinically relevant [4]. Finally, one cannot a priori extrapolate the present findings *in vitro* to a more complex *in vivo* situation. However, in a scenario in which one or several aspects of the transplantation of encapsulated islets are not successful the present data should be helpful in “problem-shooting” by minimizing the possibility of increased sensitivity to hypoxia.

## 7. Conclusions

This *in vitro* study has revealed subtle functional differences between alginate-encapsulated and free islets but none of these differences indicate that encapsulation increases susceptibility to negative effects of acute hypoxia. Indeed, the outcome of several parameters indicates better resilience towards hypoxia. This is a positive finding in relation to potential use of encapsulation for islet transplantation.

## Supplementary Material

Supplementary information on measurements of immune mediators as assayed by the Bio-Plex Panel.
Table S1 gives intra assay variations. Table S2 and Figure S1 presents information on immune mediators for which measurements came out close to the detection limit. Tables S3 and S4 compares secretion during and after the 8h hypoxic period.

## Figures and Tables

**Table 1:** Donor and donor islet characteristics.

Purity (%)	Viability (%)	GSI prior to shipment	Age (years)	BMI
87.3 ± 2.7	93.0 ± 0.6	2.1 ± 0.4	46.4 ± 4.8	30.2 ± 2.7
(80–95)	(91–95)	(1.24–3.22)	(32–59)	(22.6–40.8)

Data are mean ± SEM (range), *n* = 5.

GSI equals glucose stimulation index.

**Table 2:** Absorbance values (570 nm) representing islet viability measured by MTT.

Encapsulated islets			Non-encapsulated islets		
Normoxia	Hypoxia	Reduction by hypoxia (%)	Normoxia	Hypoxia	Reduction by hypoxia (%)
0.15 ± 0.02	0.10 ± 0.01*	33.8 ± 3.50	0.15 ± 0.02	0.09 ± 0.02*	42.9 ± 5.20

*P < 0.002 for the effect of hypoxia, *P* < 0.2 for the comparison of hypoxia-induced reduction of viability for encapsulated versus non-encapsulated islets. Data are mean ± SEM for 12 separate experiments (two-five parallels per condition), one-four experiments per donor (five donors).

**Table 3:** Effect of hypoxia on islet secreted mediators by H/N ratios (fold increase by hypoxia).

	Encapsulated islets		Nonencapsulated islets	
	Mean ± SEM	Median	Mean ± SEM	Median
IL-6	3.52 ± 0.80*	2.48	28.43 ± 24.22^∗a^	4.66
IL-9	0.97 ± 0.11	1.01	1.09 ± 0.17	0.95
IL-12	0.88 ± 0.07	0.93	0.69 ± 0.09^∗#^	0.71
IL-8	1.59 ± 0.23*	1.39	3.19 ± 0.79^∗#^	2.11
MCP-1	1.12 ± 0.09	0.98	1.40 ± 0.15^∗#^	1.31
VEGF	0.91 ± 0.06	0.88	0.70 ± 0.07^∗#^	0.72

*P < 0.001–0.05 for the effect of hypoxia, ^#^ *P* < 0.01–0.05 for the comparison of encapsulated versus non-encapsulated islets. Data are based on 11–13 single experiments (one sample per condition), one-five experiments per donor (five donors). ^a^Mean ± SEM contains one outlier value. Excluding the outlier changes mean ± SEM to 5.23 ± 1.19 and median to 4.14.

H/N equals ratio of hypoxic to normoxic conditions.
