# Supplementary material for: Alginate Microencapsulation of Human Islets Does Not Increase Susceptibility to Acute Hypoxia
Source: J Diabetes Res. 2013 Dec 1;2013:374925. doi: 10.1155/2013/374925 (PMC3864170; doi:10.1155/2013/374925)
Supplement: Supplementary file 1 — Supplementary information on measurements of immune mediators as assayed by the Bio-Plex Panel. Table S1 gives intra assay variations. Table S2 and Figure S1 presents information on immune mediators for which measurements came out close to the detection limit. Tables S3 and S4 compares secretion during and after the 8h hypoxic period. [file 374925.f1.pdf]

Supplementary information on measurements of immune mediators as assayed by the Bio-Plex Panel.

Table S1 gives intra assay variations. Table S2 and Figure S1 presents information on immune mediators for which measurements came out close to the detection limit. Tables S3 and S4 compares secretion during and after the 8h hypoxic period.

Table S1

Intra assay variations in the multiplex analysis

	CV (%) for observed concentrations		
	Mean	Median	Range
IL-6	7.02	3.88	0.16 – 33.95
IL-9	16.36	12.94	0.24 – 73.44
IL-12	13.33	11.33	0.91 – 60.12
IL-8	6.83	4.82	0.10 – 28.62
MCP-1	4.61	3.52	0.09 – 15.49
VEGF	6.98	4.76	0.30 – 27.75

Table S2

Effect of hypoxia on islet secreted mediators as measured by H/N ratios (fold increase by hypoxia)

	Encapsulated islets		Non-encapsulated islets	
	Mean ± SEM	Median	Mean ± SEM	Median
IL-10	1.37 ± 0.39	1.00	1.66 ± 0.12*	0.56
MIP-1b	1.20 ± 0.16	1.04	1.64 ± 0.47	1.18
IL-1ra	1.28 ± 0.26	1.09	1.22 ± 0.14	1.31
GM-CSF	1.33 ± 0.10*	1.18	2.09 ± 1.03	1.02
MIF	4.07 ± 1.13*	2.49	4.12 ± 1.01*	2.99

*$p < 0.05$ for the effect of hypoxia. Data represents 11-13 single experiments (one sample per condition), one-five experiments per donor (five donors). H/N equals ratio of hypoxic to normoxic conditions.

Table S3

Accumulation of mediators during 8h of hypoxia as % of secretion during total incubation period (22-26h)

	Encapsulated islets		Non-encapsulated islets	
	Normoxia	Hypoxia	Normoxia	Hypoxia
IL-6	49.5 ± 11.5	29.6 ± 11.4	55.1 ± 6.9	11.7 ± 3.5
IL-8	56.9 ± 9.5	47.6 ± 13.4	58.2 ± 8.9	30.4 ± 9.4
MCP-1	41.9 ± 8.9	35.3 ± 10.6	56.0 ± 10.3	31.5 ± 11.3
VEGF	39.0 ± 6.4	37.6 ± 8.1	41.6 ± 7.8	37.9 ± 8.2

Data are mean ± SEM of four separate experiments (one sample per condition), two experiments per donor (two donors).No significant differences could be detected.

Table S4

Secretion of mediators (pg/islet/h) after hypoxia exposure

	Secretion during	Encapsulated islets	Non-encapsulated islets
IL-6	8h of hypoxia	0.050 ± 0.028	0.017 ± 0.007[ab]
	Re-oxygenation	0.074 ± 0.035	0.116 ± 0.069
IL-8	8h of hypoxia	0.559 ± 0.285	0.153 ± 0.067[b]
	Re-oxygenation	0.232 ± 0.078	0.190 ± 0.070[b]
MCP-1	8h of hypoxia	0.251 ± 0.092	0.142 ± 0.072[b]
	Re-oxygenation	0.216 ± 0.080	0.128 ± 0.030
VEGF	8h of hypoxia	0.310 ± 0.143	0.222 ± 0.091
	Re-oxygenation	0.244 ± 0.040	0.146 ± 0.025[b]

Data are mean ± SEM of four separate experiments (one sample per condition), two experiments per donor (two donors).

[a]$p < 0.07$ for the comparison of secretion during 8h of hypoxia vs. the re-oxygenation period,

[b]$p < 0.07$ for comparison secretion by encapsulated vs. non-encapsulated islets exposed to hypoxia.

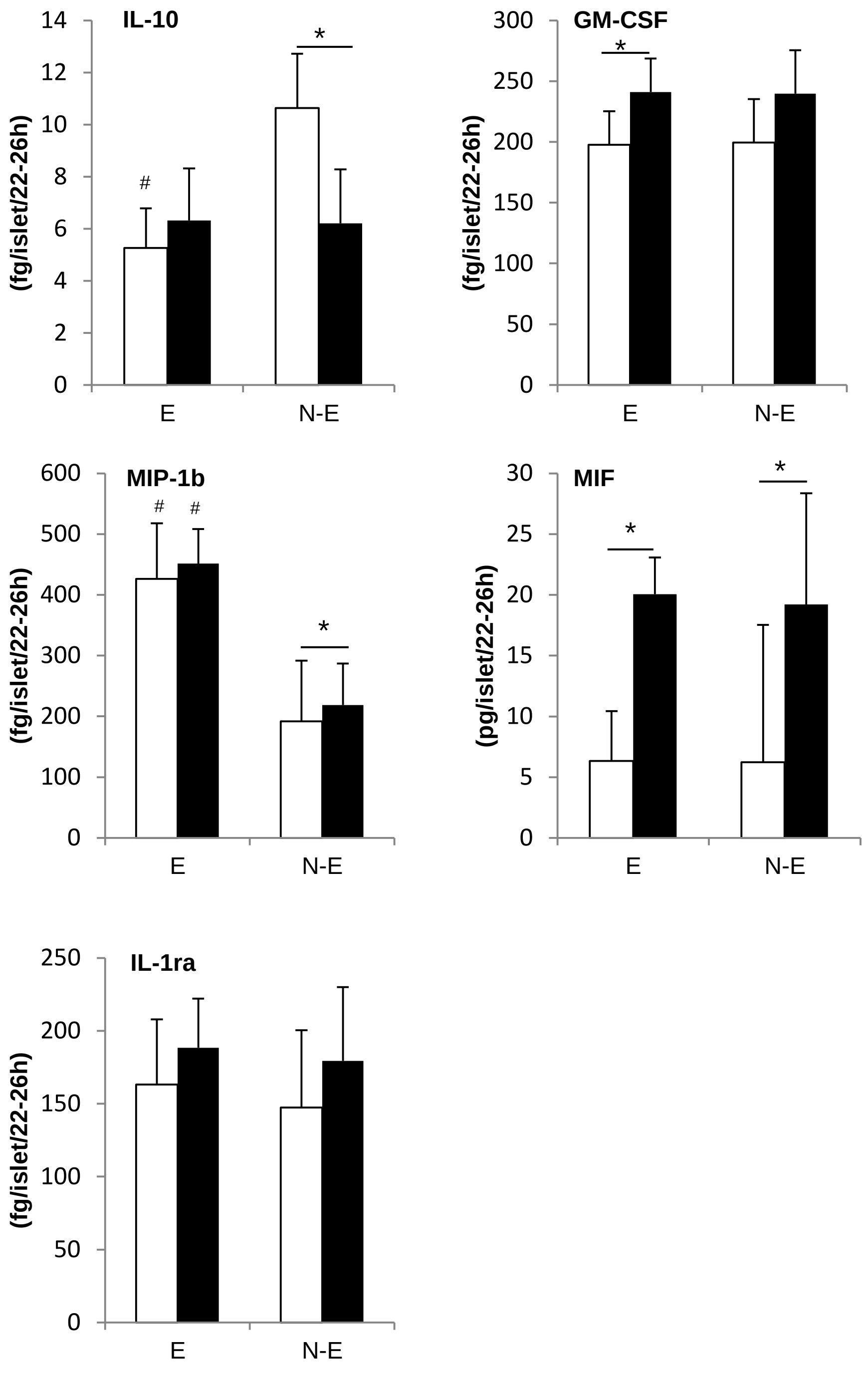

Fig S1. Secreted mediators from encapsulated (E) and non-encapsulated (N-E) islets following culture in continuous normoxia (open bars) and transient hypoxia (filled bars). *P < 0.001-0.04 for the effect of hypoxia, $^{\#}p < 0.05$ for the effect of encapsulation during normoxia. Data are

mean ± SEM of 13 separate experiments (one sample per condition), one-five experiments per donor (five donors).
